# *Akkermansia muciniphila* Metabolite Inosine Inhibits Castration Resistance in Prostate Cancer

**DOI:** 10.3390/microorganisms12081653

**Published:** 2024-08-12

**Authors:** Yao Yu, Leqian Li, Qishen Yang, Jingwen Xue, Benlin Wang, Ming Xie, Wentai Shangguan, Zhangrui Zhu, Peng Wu

**Affiliations:** Department of Urology, Nanfang Hospital, Southern Medical University, Guangzhou 510515, China; cqlpyy123@163.com (Y.Y.); doctorleqian@foxmail.com (L.L.); doctorqishen@163.com (Q.Y.); xuejwwwww@163.com (J.X.); wangbenlin01@163.com (B.W.); bioxieming@foxmail.com (M.X.); docsgwt@163.com (W.S.); m18851831561@163.com (Z.Z.)

**Keywords:** castration-resistant prostate cancer, gut microbiota, androgen deprivation therapy, *Akkermansia muciniphila*, inosine

## Abstract

Prostate cancer (PCa) is initially sensitive to androgen deprivation therapy (ADT) but ultimately develops resistance and progresses to castration-resistant prostate cancer (CRPC) with a poor prognosis. This study indicated that some PCa patients and mice were more sensitive to ADT and entered CRPC later, which was related to the gut microbiota, especially the enrichment of *Akkermansia muciniphila* (AKK). Untargeted metabolomics analysis found that serum inosine level was upregulated in the treatment-sensitive group and significantly correlated with AKK. Furthermore, we revealed that intestinal permeability and serum lipopolysaccharide (LPS) levels increased in treatment-resistant mice. LPS stimulated the upregulation of p-NF-κB p65 and AR in tumors. Supplementing AKK metabolite inosine could alleviate intestinal barrier damage and reduce serum LPS level, ultimately inhibiting castration resistance via the LPS/NF-κB/AR axis. Finally, we constructed a predictive model for CRPC combining gut microbiota and clinical information (AUC = 0.729). This study revealed the potential mechanism of gut microbiota on CRPC and provided potential therapeutic targets and prognostic indicators.

## 1. Introduction

Prostate cancer (PCa) represents a significant contributor to cancerous and socioeconomic burden worldwide [1,2]. PCa is initially sensitive to androgen deprivation therapy (ADT) [3]. However, hormone-sensitive prostate cancer (HSPC) patients gradually acquire resistance, ultimately progressing to castration-resistant prostate cancer (CRPC) with a poor prognosis [4,5]. Recently, gut microbiota has been found to be involved in the progression of PCa. Including synthesizing androgens to promote endocrine resistance [6,7,8]. However, the main reason for the progression to CRPC is the reactivation of androgen receptor (AR), which includes the aberrant amplification and/or overexpression of AR [9,10], AR mutations [11], and the generation of androgen receptor splice variants [12,13]. How gut microbiota influences CRPC deserves further exploration.

Noticeably, the time from the ADT initiation to castration-resistant progression (progression-free survival, PFS) is a key indicator reflecting the effectiveness of ADT. There are individual differences in PCa patients, and patients with longer PFS are more sensitive to ADT with better prognosis [14]. We recruited 30 CRPC patients and filtered them into the treatment-resistant (hRES) and the treatment-sensitive (hSEN) groups based on PFS. The results indicated that *Akkermansia muciniphila* (AKK) was enriched in the hSEN group. AKK is a Gram-negative resident bacterium that forms an important role in host metabolic and immune functions. Numerous studies have reported the potential clinical applications of AKK as a probiotic, particularly in chronic inflammatory diseases and cancer immunotherapy [15,16,17,18]. However, the impact of AKK on CRPC progression and its underlying mechanisms remain unclear.

Inflammation is the primary driving force behind the onset and advancement of PCa, regulating AR signaling [7]. Especially, the diminishment of androgens can induce intestinal inflammation and dysfunction [19,20]. The gut microbiota may intervene in the progression of CRPC through inflammatory pathways, requiring more laboratory evidence and clinical findings.

In this study, we found that castration resistance in PCa is related to gut microbiota composition and metabolism. AKK-specific enrichment was associated with more sensitive ADT effects in PCa patients and mice. Further investigation revealed that AKK metabolite inosine can restore intestinal dysfunction and reduce lipopolysaccharide (LPS) circulation level, thereby inhibiting castration resistance in PCa. Finally, we also constructed a predictive model for CRPC combining gut microbiota and clinical information. These findings provide preliminary evidence for the impact of gut microbiota on CRPC.

## 2. Materials and Methods

### 2.1. Cell Culture

TRAMP-C1 (ATCC CRL-2730™) and Caco-2 (ATCC HTB-37™) cell lines were purchased from Pricella Biotechnology Co., Ltd. (Wuhan, China) and cultured following ATCC guidelines. TRAMP-C1 cultured in DMEM with 0.005 mg/mL bovine insulin, 10 nM DHEA, and 10% FBS (Gibco, New York, NY, USA). Caco-2 cultured in EMEM with 20% FBS (Gibco, New York, NY, USA).

### 2.2. Bacterial Culture

*Akkermansia muciniphila* (ATCC BAA-835™) was purchased from Guangdong Microbial Culture Collection Center (Guangzhou, China) and cultured in BHI Agar/Broth (Solarbio, Beijing, China, LA0360) with 0.125% mucin (Sigma-Aldrich, Saint Louis, MO, USA, M2378) at 37 °C under anaerobic condition. Heat-killed *Akkermansia muciniphila* (dead AKK) was obtained at 100 °C for 10 min.

### 2.3. Mice

C57BL/6J mice (male, 6–8 weeks, Specific pathogen-free) were purchased from SPF Biotechnology Co., Ltd. (Beijing, China). For allograft experiments, C57BL6/N mice were challenged with 5 × 10^6^ TRAMP-C1 cells. Tumor volume was measured: V = π/6 × L × W^2^, L: the longest diameter and W: the width of the tumor. Surgical castration was performed with pentobarbital anesthesia. All experimental designs followed the protocol approved by the local Animal Care and Use Committee of the Nanfang Hospital of Southern Medical University (NFYY-2021-0572).

### 2.4. Human Study

**Figure 1:**
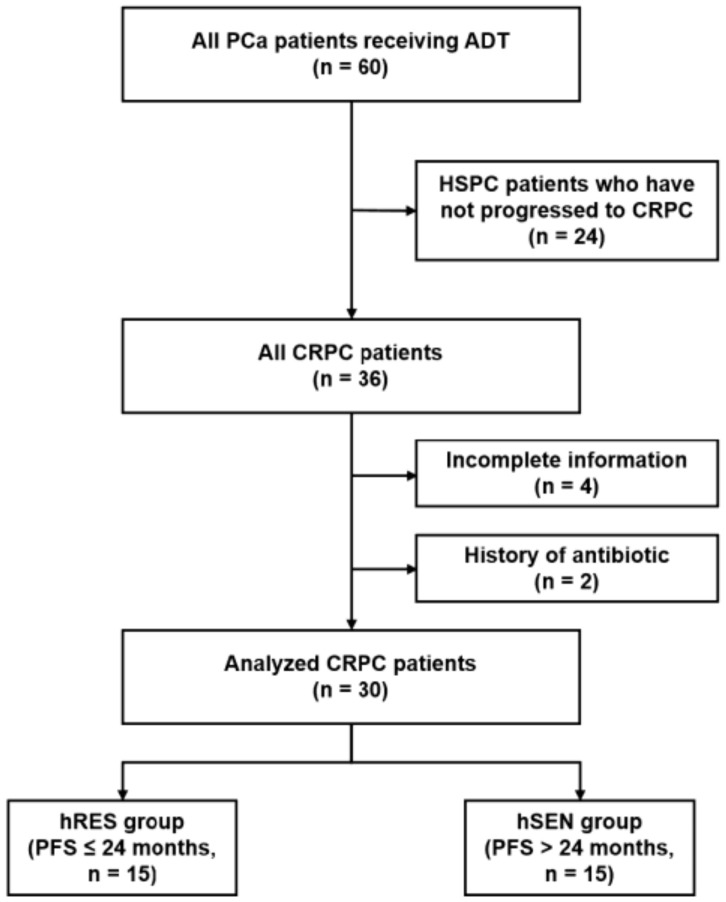
Enrollment schematic of CRPC patients. Patients were divided into the hRES group (PFS ≤ 24 months) and the hSEN group (PFS > 24 months) based on PFS.

From 2021 to 2023, 60 patients who underwent ADT were recruited from Nangfang Hospital, and the last 30 CRPC [21] patients were included in the analysis (Figure 1). Primary exclusion criteria included a history of antibiotic, probiotic, or hormone use within the preceding 3 months, any acute or chronic illness, history of severe organic diseases. Age, body mass index (BMI), TNM stage, Gleason score, and prostate-specific antigen (PSA) level were considered as patients’ baseline measurements. The time to CRPC was served as PFS. The minimum PSA value after ADT was defined as nPSA [22,23]. This study has been approved by the Ethics Committee of Nanfang Hospital, Southern Medical University (NFEC-2020-123).

### 2.5. Fecal Microbiota Transplantation Experiment 

Fecal samples from the donor mice (RES and SEN group) were obtained in aseptic centrifugal tubes, respectively, and resuspended in PBS (125 mg/mL). The supernatants, after centrifugation for 1 min at 1000 g, were saved in separate 1.5 mL tubes for subsequent microbiota transplantation. Before the FMT experiment, the receiver mice were orally administered a cocktail of antibiotics, including vancomycin (100 mg/kg), neomycin sulfate (200 mg/kg), metronidazole (200 mg/kg), and ampicillin (200 mg/kg) to clear the gut microbiota [24].

### 2.6. FITC-Dextran Permeability Experiment

In vivo

FITC-Dextran solution (Sigma, Shanghai, China) was administered to mice (0.6 mg/kg), serum was collected four hours later, and the FITC-Dextran content in serum was measured by microplate fluorescence reader (Infinite^®^ M1000 PRO, TECAN, Männedorf, Switzerland). Permeability was calculated by normalizing to the read of the control group.

In vitro

Inoculate cells at the Transwell system until forming a tight monolayer. 200 µL of 10 µM FITC-Dextran was added to the apical side, and after four hours, the content of FITC-Dextran on the basal side was measured using a microplate fluorescence reader.

### 2.7. Trans-Epithelial Electrical Resistance (TEER)

Measuring the TEER value of the monolayer of Caco-2 cells can reflect the barrier function of the cells. Inoculate cells at the Transwell system until forming a tight monolayer along with TEER reaching 500 mΩ. The changes in TEER were determined by the MILLICELL-ERS volt-ohm meter system (Millipore, Burlington, MA, USA), and the results represent the average of measurements taken at three different locations.

### 2.8. ELISA

LPS, interleukin-6 (IL-6), tumor necrosis factor (TNF-α), interleukin-1β (IL-1β), inosine, and testosterone concentrations were detected by ELISA Kit (MEIMIAN, Yancheng, China).

### 2.9. Gene Expression Analysis

Total RNA was collected by RNA Extraction Kit (Foregene, Chengdu, China). cDNA was obtained by the reverse transcript enzyme HiScript III Super Mix (Vazyme, Nanjing, China). The qPCR reactions were proceeded by ChamQ SYBR Master Mix (Vazyme, Nanjing, China). Quantify the relative level of target genes using the 2^−ΔΔCT^ methods with detailed primer sequences in Table S1.

### 2.10. Protein Expression Analysis

The protein was collected by RIPA lysis buffer (Epizyme Biomedical Technology Co., Ltd., Shanghai, China) with a 1% protease inhibitor (Meilunbio, Dalian, China). Subsequently, the concentration was measured by a BCA assay kit (Beyotime, Shanghai, China). Adjust the concentration to 2 μg/μL and mix with loading buffer (Fdbio, Hangzhou, China) at 100 °C for 15 min to denature the protein. Proteins were separated by molecular weight using 12% SDS-PAGE. Afterward, Proteins were transferred to the PVDF membrane through electrophoresis (Merck Millipore, Burlington, MA, USA). Incubate the blocked membrane with primary antibody at 4 °C for 12 h. And then incubated with a secondary antibody at 25 °C for 1 h. The membrane was observed by enhanced chemiluminescence (Biosharp, Hefei, China). Quantify the relative level of the target protein using Image J (V1.53n) [24]. The primary antibody used in the Western blot experiment is as follows: ZO-1 (1/1000, Abcam, Massachusetts, USA), Occludin (1/1000, Abcam, Massachusetts, USA), AR (1/2000, Abmart, Shanghai, China), p-NF-κB p65 (1/1000, Abmart, Shanghai, China), NF-κB p65 (1/5000, Abmart, Shanghai, China), and β-actin (1/1000, Ray Antibody Biotech, Beijing, China).

### 2.11. Histological Staining and Analysis

The tissue was fixed, embedded, and prepared into 4 µm thick slices. The slices were performed antigen repair by sodium citrate solution and then blocked in 3% H_2_O_2_ at 25 °C for 30 min. Incubate with primary antibody at 4 °C for 12 h, and then incubate with secondary antibody at 25 °C for 1 h. Finally, diaminobenzidine (Maixin, Fuzhou, China) was applied for immunohistochemical reactions and then stained with hematoxylin. The positivity ratio of positive cells (Ki-67, 1/1000, Proteintech, Wuhan, China) or the mean density (ZO-1, 1/500, Occludin, 1/500, Abcam, Waltham, MA, USA) of each sample was statistically analyzed by randomly selecting five fields of view. All images were scanned by a Nano-Zoomer Digital slide scanner and captured at ×200 or ×400 with an NDP: View2 Plus Image viewing software (V1.8.1) (Hamamatsu Photonics, Shizuoka, Japan).

### 2.12. 16. S rDNA Analysis

Extract DNA by DNA Stool Mini Kit (TIANGEN, Beijing, China) [25,26] and then amplify the V3-V4 region via PCR. Data analysis was performed on the Illumina platform. Alpha diversity was computed by QIIME (V1.9.1) [27]. NMDS of unweighted unifrac distances and PCoA were calculated using R (V2.5.3). Differentially, taxa were identified using LEfSe software (V1.0) [28].

### 2.13. Untargeted Metabolomic Analysis

Mix 50 μL of serum with 200 μL of 0 °C pre-cooled methanol; ultrasonic extraction was performed at 0 °C for 10 min × 3 and left to stand overnight at −20 °C. Collect supernatant by centrifugation at 4 degrees Celsius and 12,000 rpm for 20 min. Perform LC/MS analysis and information extraction using the UPLC-TripleTOF system (AB SCIEX). At 40 °C, 0.40 mL/min, 20 μL begins to inject samples with water (A) and acetonitrile (B). Adjust the ratio and time of the two inputs according to the following procedure (initial to 0–2 min: 2% B, 2–5 min: 2–60% B, 5–8 min: 60–95% B, 8–10 min: 95–100% B, 10–12.5 min: 100% B, 12.5–12.6 min: 100%–2% B, 12.6–15 min: 2–0% B) Using positive and negative ion scanning to collect data, with injection and collision voltage of 1.0 kV, 40 V, and 6 eV, respectively. Obtain a data matrix using Progenesis QI (Waters Corporation, Milford, MA, USA) and match it with the metabolic database. Metabolite and microbial integrated association analysis was calculated using R (V1.8.4) and then generated by Majorbio Cloud Platform.

### 2.14. Statistical Analysis

Statistical comparisons were conducted by either one-way analysis of variance (ANOVA) or Student’s *t*-test. The results were represented as means ± standard deviation and processed by GraphPad Prism software (version 8.0). Univariable and multivariable Cox regression models were utilized to assess the effects of individual or multiple variables on PFS.

## 3. Results

### 3.1. Differences in Gut Microbiota among hRES and hSEN PCa Patients

Patients with longer PFS are more sensitive to ADT with better prognosis [14]. Thirty CRPC patients were classified into the treatment-resistant group (hRES ≤ 24 months) and the treatment-sensitive group (hSEN > 24 months) according to PFS (Figure 1). As demonstrated in Table 1, patients’ baseline characteristics were collected. Notable divergences in PFS, age, BMI, and nPSA were found between the hRES and hSEN groups.

**Figure 2:**
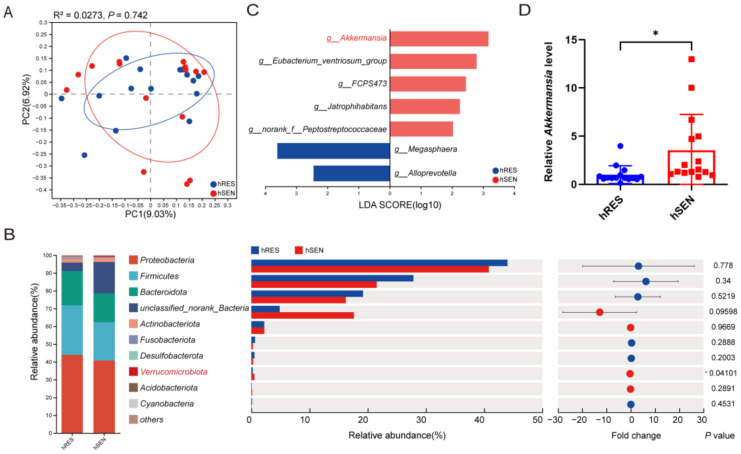
Differences in gut microbiota among hRES and hSEN PCa patients. (**A**) Beta diversity between hRES and hSEN groups of PCoA. *n* = 15. (**B**) Differences in community abundance at the phylum level between hRES and hSEN groups. *n* = 15. (**C**) LEfSe analysis with LDA > 2 between hRES and hSEN groups. *n* = 15. (**D**) Relative abundance of *Akkermansia* in hRES and hSEN groups measured by qPCR. *n* = 15. Data were expressed as the mean ± SD, * *p* < 0.05. PCoA, Principal coordinate analysis; LDA, Linear discriminant analysis.

Subsequently, fecal specimens were collected for 16S rDNA sequencing. The results revealed no significant disparities between the hRES and hSEN groups of patients in microbiota diversity (Figure S1A and Figure 2A). The relative abundances of *Verrucomicrobiota* exhibited a significant increase at the phylum level in the hSEN group (Figure 2B). LEfSe demonstrated that *Akkermansia* and *Eubacterium ventriosum* were higher in the hSEN group, and *Megasphaera* and *Alloprevotella* were higher in the hRES group (Figure 2C). Subsequently, qPCR data validated the enrichment of *Akkermansia* in patients of the hSEN group (Figure 2D).

### 3.2. Gut Microbiota Influenced the Resistance to ADT against PCa

**Figure 3:**
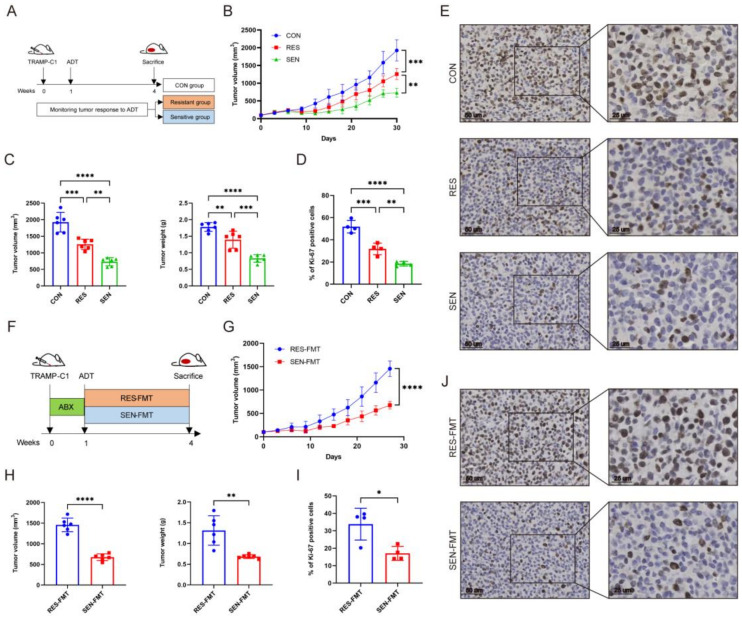
Gut microbiota influenced the resistance to ADT against PCa. (**A**) The experimental schematic. Castration surgery was performed on mice with TRAMP-C1 subcutaneous tumor models 1 week later. (**B**,**C**) Tumor volume growth curve, volume, and mass. *n* = 6. (**D**,**E**) Tumor Ki67-positive cell percentage and immunohistochemical image. Scale bars represent 25 mm. *n* = 4. (**F**) The experimental schematic. Mice with subcutaneous tumor models were pre-treated with ABX and castration surgery, followed by fecal microbiota transplantation from the RES and SEN groups mice for 3 weeks. (**G**,**H**) Tumor volume growth curve, volume, and mass. *n* = 6. (**I**,**J**) Tumor Ki67-positive cell percentage and immunohistochemical image. Scale bars represent 25 mm. *n* = 4. Data were expressed as the mean ± SD, * *p* < 0.05, ** *p* < 0.01, *** *p* < 0.001, **** *p* < 0.0001.

Next, the PCa syngeneic transplantation model is established through the mouse PCa cell line TRAMP-C1 subcutaneous injection. Surgical castration was performed one week later, and tumor volume was conducted to evaluate the tumor’s response to ADT. Based on the difference in tumor growth rate after ADT, mice were classified into the treatment-resistant (RES) group and the treatment-sensitive (SEN) group (Figure 3A). The SEN group exhibited a slower growth rate, smaller tumor volume and mass, and a lower proportion of Ki67-positive cells compared with the RES group (Figure 3B–E). The serum testosterone level was similar between the two groups (Figure S2A).

To further validate the tangible impact of gut microbiota on ADT, we then collected feces from both groups for their transplantation into recipient mice (Figure 3F). Compared to mice receiving feces from the RES group, those receiving feces from the SEN group exhibited significantly reduced tumor growth rate, volume, and mass and significantly decreased Ki67-positive cell ratio (Figure 3G–J). Additionally, there were no differences in serum testosterone levels (Figure S2B), ruling out the possible influence of serum testosterone. Therefore, these results preliminarily indicate that gut microbiota influenced the resistance to ADT against PCa.

### 3.3. AKK Was Associated with Treatment Sensitivity to ADT

**Figure 4:**
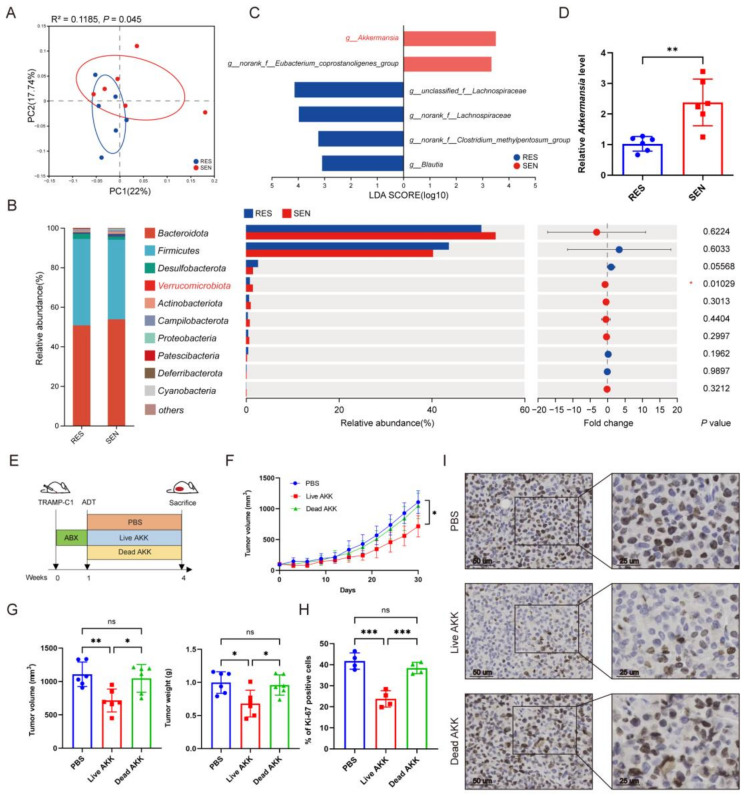
AKK was associated with treatment sensitivity to ADT. (**A**) Beta diversity between RES and SEN groups of PCoA. *n* = 6. (**B**) Differences in community abundance at the phylum level between RES and SEN groups. *n* = 6. (**C**) LEfSe analysis with LDA > 3 between RES and SEN groups. *n* = 6. (**D**) Relative abundance of *Akkermansia* in RES and SEN groups measured by qPCR. *n* = 6. (**E**) The experimental schematic. Mice with subcutaneous tumor models were pre-treated with ABX and castration surgery, then administered with live AKK, dead AKK, or PBS for 3 weeks (2 × 10^8^ CFU). (**F**,**G**) Tumor volume growth curve, volume, and mass. *n* = 6. (**H**,**I**) Tumor tissue Ki67-positive cell percentage and immunohistochemical image. Scale bars represent 25 mm. *n* = 4. Data were expressed as the mean ± SD, ns = no significance, * *p* < 0.05, ** *p* < 0.01, *** *p* < 0.001. PCoA, Principal coordinate analysis; LDA, Linear discriminant analysis; AKK, *Akkermansia muciniphila*.

The variations in mice gut microbiota were observed through 16S rDNA sequencing, consistent with our findings in patients. Alpha diversity analysis showed that the gut bacterial community between the RES group and SEN group was similar (Figure S1B). PCoA revealed distinct clustering and separation of microbial composition between the two groups (Figure 4A). The relative abundance of *Verrucomicrobiota* significantly increased at the phylum level in the SEN group (Figure 4B,C). LEfSe demonstrated that *Akkermansia* and *norank_f_Eubacterium* were significantly higher in the SEN group at the genus level (Figure 3D). Subsequently, qPCR results confirmed that *Akkermansia* was enriched in the SEN group mice (Figure 4E).

These results verified intestinal *Akkermansia* enrichment in the treatment-sensitive patients and mice, indicating an underlying relationship between increased AKK abundance and treatment sensitivity to ADT. To further validate the physiological function, AKK was administered to mice to explore whether it could influence the effectiveness of ADT in PCa (Figure 4G). Compared to the PBS group, live AKK could significantly enhance ADT, resulting in a slower growth rate, smaller tumor volume and mass, and a lower proportion of Ki67-positive cells (Figure 4H–I). Notably, heat-killed AKK had no effect. Therefore, we speculate that AKK may generate bioactive metabolites to exert an effect.

### 3.4. The Elevated Inosine Synthesis in SEN Group Was Associated with AKK

**Figure 5:**
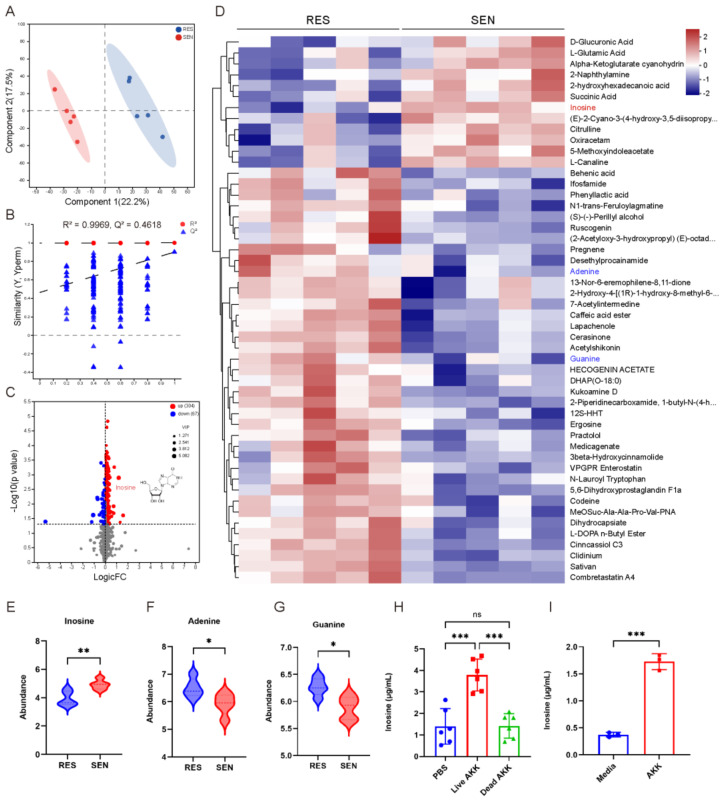
The elevated inosine synthesis in the SEN group was associated with AKK. (**A**) the PLS-DA analysis between RES and SEN groups. Each point represented a sample, and the elliptical range represented the 95% confidence interval. *n* = 5. (**B**) Permutation testing of the PLS-DA model. (**C**) Volcano plot of metabolite between RES and SEN group. (**D**) Cluster heatmap of differential metabolites in untargeted metabolomics. *n* = 5. (**E**–**G**) Abundance level of inosine, adenine, and guanine in RES and SEN group mice. The width of the violin plot represented kernel density, indicating the distribution shape of the data. *n* = 5. (**H**) Inosine level in mice administered PBS, live AKK, or dead AKK. *n* = 6. (**I**) Inosine level in AKK culture supernatant. *n* = 3. Data were expressed as the mean ± SD, ns = no significance, * *p* < 0.05, ** *p* < 0.01, *** *p* < 0.001. PLS-DA, Partial least squares Discriminant Analysis.

Subsequently, we performed untargeted metabolomics on the serum of RES and SEN group mice. PLS-DA analysis showed a clear distinction in the metabolite composition of the two groups (Figure 5A), and the PLS-DA model was stable and reliable by cross-validation (Figure 5B, R^2^ = 0.9969, Q^2^ = 0.4618). There were a total of 304 increased metabolites and 67 decreased metabolites in the SEN group (Figure 5C and Figure S3A, VIP > 1, *p* < 0.05). Especially, there were significant changes in purine metabolites (Figure 5D), inosine (Figure 5E), adenine (Figure 5F), and guanine (Figure 5G). Subsequently, we conducted an integrated correlation analysis between metabolites and bacterial abundance (Figure S3B) and found that AKK was positively correlated with inosine (r = 0.606, *p* = 0.04) and negatively correlated with adenine (r = −0.764, *p* = 0.01) and guanine (r = −0.499, *p* = 0.14). Additionally, we measured the inosine in the serum of AKK-intervened mice and the AKK culture supernatant. The serum inosine levels in the PBS, live AKK, and dead AKK groups were 1.203 (0.651–2.263), 3.783 (3–4.565), and 1.557 (0.793–1.878) μg/mL, respectively. The presence of inosine in plasma increased significantly with live AKK treatment. Specifically, the plasma inosine level increased by approximately 2.58 μg/mL (from 1.203 to 3.783 μg/mL) in the live AKK group. In contrast, the dead AKK group showed no significant increase (from 1.203 to 1.557 μg/mL). These indicated that AKK could promote inosine synthesis both in vivo (Figure 5H) and in vitro (Figure 5I).

### 3.5. Intestinal Barrier Dysfunction and Elevated LPS Promoted Castration Resistance

**Figure 6:**
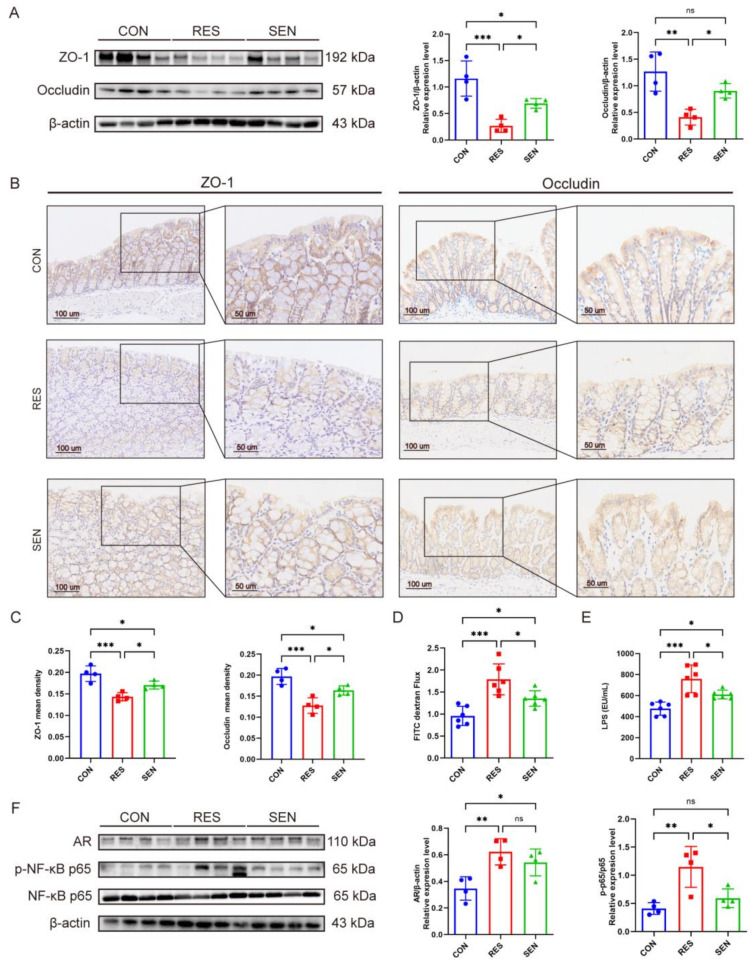
Intestinal barrier dysfunction and elevated LPS promoted castration resistance. (**A**–**C**) The protein levels of ZO-1 and Occludin in the colon of RES and SEN groups by Western blotting and immunohistochemistry. Scale bars represent 50 mm. *n* = 4. (**D**,**E**) FITC-dextran flux and serum LPS level in RES and SEN groups. *n* = 6. (**F**) The protein levels of AR, p-NF-κB p65, and NF-κB p65 in tumor of RES and SEN groups by Western blotting, *n* = 4. Data were expressed as the mean ± SD, ns = no significance, * *p* < 0.05, ** *p* < 0.01, *** *p* < 0.001.

AKK is well known for its capacity to improve inflammatory responses and restore intestinal barrier function [29]. Intestinal-derived LPS is a key inflammatory inducer that can promote tumor progression [30,31,32]. Therefore, we speculated that the resistance to ADT may be related to intestinal inflammation. The intestinal barrier disorders were observed after ADT and the RES group mice exhibited lower levels of ZO-1 and Occludin at both transcription and expression in the colon than the SEN group (Figure 6A–C and Figure S4A). Compared to the SEN group, the RES group exhibited higher intestinal epithelial permeability in FITC-dextran flux (Figure 6D) and serum LPS level (Figure 6E). In addition, the content of IL-6, TNF-α, and IL-1β were notably higher in circulation (Figure S4B). Correspondingly, tumors in the RES group showed significantly higher levels of NF-κB phosphorylation and increased transcription and expression of AR (Figure 6F and Figure S4C). In summary, intestinal barrier dysfunction leads to an increase in serum LPS, and LPS stimulation of NF-κB phosphorylation and AR upregulation may promote resistance to ADT.

### 3.6. The Metabolite Inosine Alleviated Intestinal Barrier Damage and Inhibited Castration Resistance

**Figure 7:**
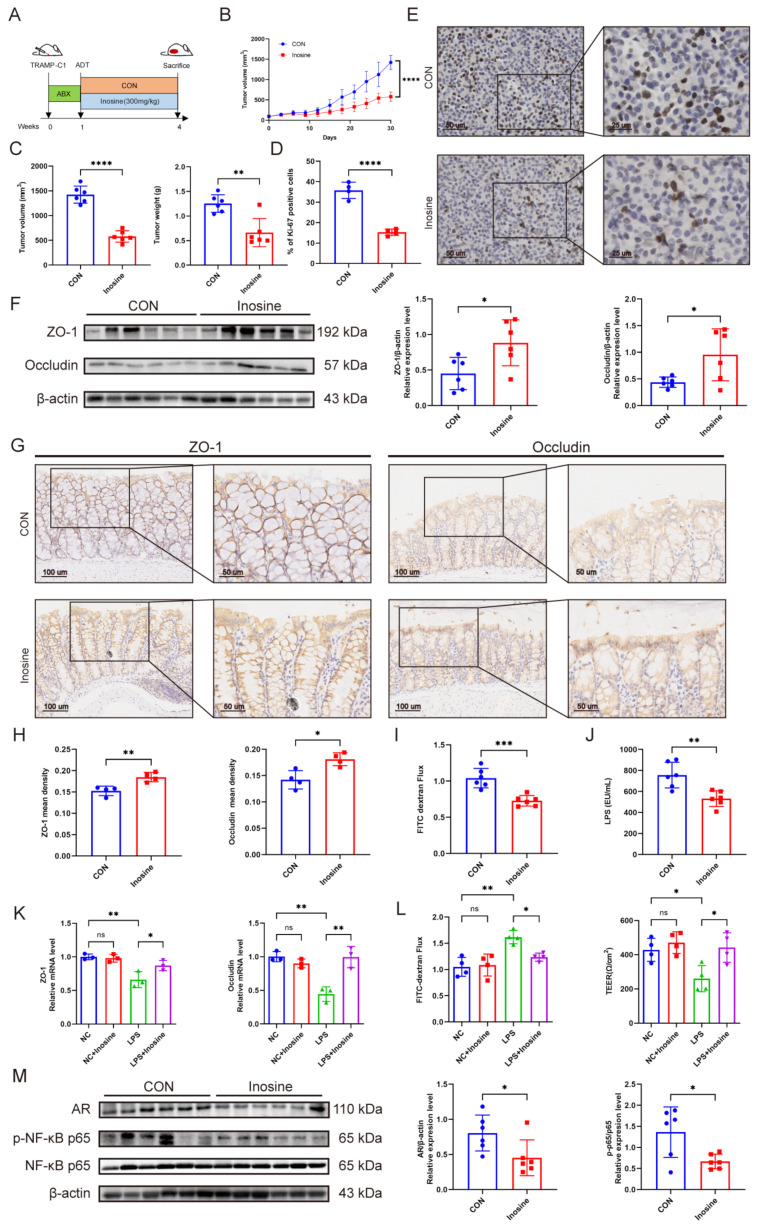
The metabolite inosine alleviated intestinal barrier damage and inhibited castration resistance. (**A**) The experimental schematic. Mice with subcutaneous tumor models were pretreated with ABX and castration surgery, then administered with inosine for 3 weeks (300 mg/kg). (**B**,**C**) Tumor volume growth curve, volume, and mass. *n* = 6. (**D**,**E**) Tumor tissue Ki67-positive cell percentage and immunohistochemistry image. Scale bars represent 25 mm. *n* = 4. (**F**–**H**) The protein levels of ZO-1 and Occludin in the colon of mice administered with inosine by Western blotting and immunohistochemistry. Scale bars represent 50 mm. *n* = 4–6. (**I**,**J**) FITC-dextran flux and serum LPS level in mice administered with inosine. *n* = 6. (**K**) The transcriptional levels of ZO-1 and Occludin in Caco-2 cells after LPS (5 μg/mL) stimulation and inosine (2 mM) intervention by qPCR. *n* = 3. (**L**) FITC-dextran flux and TEER of Caco-2 cells after LPS stimulation and inosine intervention. *n* = 4. (**M**) The protein level of AR, p-NF-κB p65, and NF-κB p65 in tumors of mice administered with inosine by Western blotting. *n* = 6. Data were expressed as the mean ± SD, ns = no significance, * *p* < 0.05, ** *p* < 0.01, *** *p* < 0.001, **** *p* < 0.0001.

Oral administration of inosine (300 mg/kg) could significantly enhance the effect of ADT, accompanied by a slower growth rate, lower tumor volume and mass, and a Ki67 positive cell ratio (Figure 7A–E). Specifically, supplementing with inosine could significantly mitigate the loss of ZO-1 and Occludin of the intestinal barrier (Figure 7F–H and Figure S5A) while reducing intestinal permeability and serum LPS level (Figure 7I,J). The circulation content of IL-6, TNF-α, and IL-1β in mice supplemented with inosine was significantly reduced (Figure S5B). Furthermore, intervention with inosine (2 mM) in the human colonic adenocarcinoma cell line Caco-2 in vitro revealed that inosine could restore the downregulation transcription level of ZO-1 and Occludin induced by LPS (Figure 7K). Inosine intervention alleviated the increase in FITC-dextran flux and the decrease in TEER caused by LPS (Figure 7L), as well as the elevation of IL-6 in the culture supernatant (Figure S5C). Further results suggested that supplementing with inosine could significantly reduce tumor NF-κB phosphorylation level and AR transcription and expression level (Figure 7M and Figure S5D). These results indicated that inosine could inhibit castration resistance by alleviating intestinal barrier damage and decreasing serum LPS levels.

### 3.7. Predictive Model for CRPC Based on Gut Microbiota

**Figure 8:**
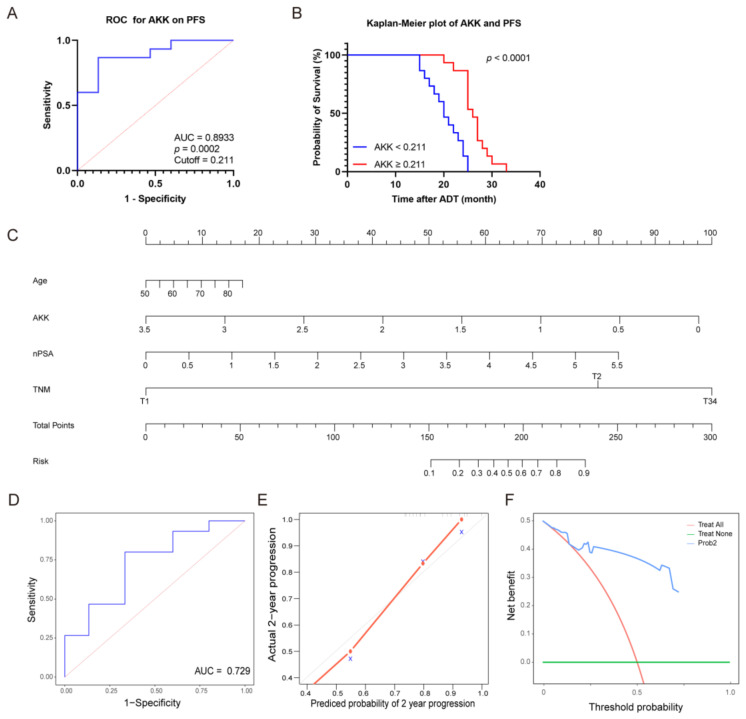
Predictive model for CRPC based on gut microbiota. (**A**,**B**) ROC analysis and Kaplan–Meier plot for AKK on PFS. The diagonal line represented the baseline of random guessing, the blue line respresented ROC curve. (**C**) Nomogram of the 2-year CRPC progress predictive model. AKK calibrated by log2 (relative abundance + 1). (**D**) ROC analysis of the nomogram. (**E**) Calibration curves of the nomogram. the diagonal line represented perfect calibration, the red line represented Calibration curve. Each point on the calibration curve corresponded to a specific predicted probability bin. (**F**) Decision curve analysis of the nomogram. Data were expressed as the mean ± SD. ROC, receiver operating characteristic; AKK, *Akkermansia muciniphila*; PFS, progression-free survival; nPSA, the nadir of prostate-specific antigen; CRPC, castration-resistant prostate cancer.

Next, we investigated the contribution of AKK on the prognosis of PCa patients. The ideal cutoff value for AKK relative abundance about PFS was 0.211% by ROC analysis (AUC = 0.8933, Figure 8A). There was a significant correlation between low AKK and poor PFS by Kaplan Meier survival analysis (Figure 8B). Next, we performed a univariate COX regression analysis on AKK and clinical indicators and found significant differences in Age, TNM, nPSA, and AKK (Table 2, Figure S6). All significant variables (*p* < 0.05) of univariate COX were considered for inclusion in the subsequent multivariate Cox regression analysis. The outcomes revealed that ultimately, solely nPSA ≥ 0.346 ng/mL (HR 2.58, 95% CI 1.28–5.21) was identified as the independent prognostic indicator for 2-year CRPC progression (Table 3). In light of COX regression analysis, we developed a nomogram plot for predicting 2-year CRPC progression, including Age, AKK value, nPSA level, and TNM stage, which all showed significant differences (Figure 8C). By aligning the patient’s specific values for these indicators with the corresponding points on the scale and summing them, clinicians could determine the total points and use the bottom risk scale to find the corresponding probability of CRPC progression within 2 years. The C-index was 0.873, and the AUC was 0.729 (Figure 8D), indicating good predictive performance of the model. The calibration curve showed a consistent probability of 2-year CRPC progression (Figure 8E). The DCA curve confirmed that the model had a significant positive net clinical benefit (Figure 8F). In summary, we proposed a prediction model for CRPC incorporating the gut microbiome and clinical data.

## 4. Discussion

The resistance mechanism of CRPC is quite complex, and there is currently no definite conclusion. The contribution of gut microbiota is a new potential perspective. This study found that prostate cancer patients and mice who were more sensitive to ADT had unique gut microbiota composition, especially the enrichment of AKK. We elucidated the key role of AKK in inhibiting castration resistance in PCa. Specifically, the inosine produced by AKK reduced the entry of LPS into the circulation by protecting the intestinal barrier. LPS activated NF-κB and upregulated AR to accelerate the progression of CRPC. Finally, we developed a reliable predictive model for CRPC, combining AKK and clinical data. These results revealed that AKK and inosine served as modulators of CRPC, providing potential therapeutic targets and prognostic indicators for CRPC.

Furthermore, previous research has revealed the involvement of gut microbiota in the absorption and metabolism of androgens, underling their vital functions in various disease processes [19,33,34]. Pernigoni et al. discovered an enrichment of tumor-associated bacteria in patients with CRPC. These bacteria could convert androgen precursors into active androgens, consequently fostering castration resistance [6].In addition, Hsiao et al. proposed that gut bacteria involved in androgen degradation metabolism might serve as targets for PCa treatment [35]. These studies focused on the disturbance and differences in microbiota before and after ADT. Differently, our study focused on the differences in treatment sensitivity among different individuals after ADT. No differences were found in serum testosterone levels and androgen-producing bacterial communities between the two groups of mice in our study. Tang et al. reported that supplementing inosine can promote testosterone secretion in the testes [36]. Although our study used a castrated mouse model, the effects of inosine on other endocrine organs still required further investigation.

Androgen was crucial in intestinal homeostasis, including modulating gastrointestinal nerve function affecting irritable bowel syndrome [20], inhibiting inflammation responses of specific gastric lymphocyte subpopulations [37], and regulating the influence of stromal cells on intestinal epithelium [38]. Our study demonstrated that intestinal permeability and inflammation levels increased after ADT. It should be noted that in PCa, besides tumor progression, the increase in inflammation level could also be attributed to tumor cell death induced by ADT [39].

As is well known, AKK has critical therapeutic benefits for intestinal barrier homeostasis. AKK has been widely reported to protect the intestinal barrier by secreting short-chain fatty acids [40,41,42]. Our study found that AKK derived inosine also protected intestinal barrier function. Consistent with previous studies, inosine may mediate the barrier regulatory effect of intestinal glial cells through the adenosine A2A receptor [43,44]. Safae et al. found that oral administration of AKK could ameliorate acquired immune deficiency to delay the growth of PCa [13]; Daisley et al. found that abiraterone acetate could promote the growth of AKK and enhance microbial VK2 synthesis to suppress PCa growth [14]. Inosine combined with immunotherapy could enhance the anti-tumor ability of T cells in colorectal cancer and bladder cancer. However, it was worth noting that supplementing inosine alone had no anti-tumor effect [45]. In this study, the intestinal microbial metabolite inosine reduced the stimulating effect of LPS on tumor cells by alleviating intestinal inflammation. The potential mechanism of inosine on tumors needed further research.

In this study, the intestinal barrier damage after ADT led to LPS entry into the circulation, LPS translocation to tumor stimulated NF-κB phosphorylation, and upregulation of AR, ultimately promoting the progression of CRPC. LPS, as a key inflammatory stimulatory factor, has a promoting effect on the proliferation and migration of various tumors [31,46,47]. Research reported that antibiotic exposure could increase the progression of LPS-stimulated RM-1 and DU-145 androgen-independent PCa cells [7]. The differences in LPS levels were attributed to variations in treatment response following ADT in this study. Furthermore, this study focused on the impact of LPS on androgen receptor activation in PCa. The NF-κB signaling pathway is crucial in the occurrence and progression of CRPC. NF-κB and AR response element-binding sites exhibit proximity and overlap. Following the activation of lipopolysaccharide (LPS), NF-κB activates the AR promoter, significantly upregulating the transcription and protein level of AR. This process facilitates AR transactivation and cell proliferation [48,49,50,51,52].

PSA decline and PSA response could serve as conventional yet practical prognostic factors for predicting patients’ biochemical recurrence outcomes [53]. Among these, nPSA has been extensively documented as a marker for forecasting disease advancement, response to treatment, and survival probabilities in HSPC [54,55]. In this study, Elevated nPSA (≥0.346 ng/mL) was associated with shorter PFS, consistent with former studies [55,56]. In particular, our study found that the lower abundance of AKK (<0.211%) was related to poor PFS. However, only nPSA was the independent risk factor for PFS in multivariable analysis, possibly due to a small sample size or collinearity between variables. The detection of gut microbiota has the advantages of non-invasive and convenient methods, although its predictive potential for CRPC still needs to be validated by larger-scale prospective clinical trials.

Previous reports have shown that AKK abundance could predict clinical benefits in immunotherapy for non-small cell lung cancer [57]. In this study, we provided a predictive model based on AKK abundance and clinical information, demonstrating good predictive ability (AUC = 0.729). However, due to the limitation of a single institution and the deficiency of clinic cases, selection bias might be a potential concern. Another potential limitation of our study was employing the progression to CRPC as the outcome measure. We chose this endpoint because it held biological and clinical significance. Biochemical recurrence in PCa indicated treatment failure with ADT, and the time to castration resistance best demonstrated the effectiveness of ADT treatment [21,22]. Furthermore, our study had not yet reached the survival endpoint, and incorporating analyses for both PFS and overall survival might yield more comprehensive findings.

## 5. Conclusions

In summary, we found that the increased AKK abundance and serum inosine level were associated with the more sensitive response to ADT in PCa. AKK secreted inosine to alleviate intestinal barrier damage and reduce serum LPS level, thereby inhibiting castration resistance through the LPS/NF-κB/AR axis. Furthermore, we established a predictive model for CRPC combining AKK and clinical indicators. In summary, this study revealed the pathological mechanism and provided potential therapeutic targets and prognostic indicators for CRPC.

## Figures and Tables

**Table 1:** Baseline characteristics.

Clinical Data	Number (%), Median (IQR)			*p* Value
	Total	hRES Group	hSEN Group	
Number of patients	30	15	15	
PFS (months)	24.5 (20–25.75)	20 (17.5–22)	26 (25–27.5)	<0.001
Age (years)	70 (65–74)	72 (68–78.5)	69 (63–72)	0.033
BMI (kg/m^2^)	23.47 (23.03–24.59)	23.25 (22.67–23.62)	24.46 (23.27–25.42)	0.026
TNM				
T1 + 2	17 (56.7%)	5	12	0.0253
T3 + 4	13 (43.3%)	10	3	
N0	22 (73.3%)	10	12	0.6817
N1	8 (26.7%)	5	3	
M0	18 (60%)	10	8	0.7104
M1	12 (40%)	5	7	
Gleason score				0.0996
G1 (3 + 3)	4 (13.3%)	1	3	
G2 (3 + 4)	5 (16.7%)	2	3	
G3 (4 + 3)	2 (6.7)	2	0	
G4 (8)	6 (20%)	1	5	
G5 (9,10)	13 (43.3%)	9	4	
PSA diagnosis (ng/mL)	15.66 (11.26–38.83)	17.11 (11.49–40.95)	14.8 (11.38–29.34)	0.339
nPSA (ng/mL)	0.27 (0.13–1.21)	1.23 (0.549–2.15)	0.13 (0.08–0.16)	<0.001

hRES group (PFS ≤ 24) and hSEN group (PFS > 24). PFS, progression-free survival; BMI, body mass index; nPSA, the nadir of prostate-specific antigen.

**Table 2:** Univariable analysis of PFS.

Variable	PFS	
	HR (95% CI)	*p* Value
Age (years)	1.1 (1–1.2)	0.05
BMI (kg/m^2^)	0.77 (0.57–1)	0.087
TNM	2.5 (1.3–4.8)	0.0068
Gleason	1.3 (0.87–1.9)	0.22
PSA diagnose (ng/mL)	1 (1–1)	0.82
nPSA (ng/mL)	3 (1.8–5)	0.00003
AKK abundance (%)	0.011 (0.00013–0.91)	0.046

PFS, progression-free survival; BMI, body mass index; nPSA, the nadir of prostate-specific antigen; AKK, *Akkermansia muciniphila.*

**Table 3:** Multivariate analysis of PFS.

Variable	PFS	
	HR (95% CI)	*p* Value
Age (years)	1.04 (0.96–1.13)	0.319
TNM	1.89 (0.88–4.00)	0.102
nPSA (ng/mL)	2.39 (1.37–4.15)	0.002
AKK abundance (%)	0.30 (0.02–3.86)	0.355

PFS, progression-free survival; nPSA, the nadir of prostate-specific antigen; AKK, *Akkermansia muciniphila.*

## Data Availability

The raw data has been archived in the Genome Sequence Archive database at the BIG Data Center. (CRA015343 and CRA015360).

## Supplementary Materials

The following supporting information can be downloaded at https://www.mdpi.com/article/10.3390/microorganisms12081653/s1, Figure S1: Alpha diversity of the gut microbiota in mice and patients; Figure S2: Serum testosterone level in mice; Figure S3: Metabolite VIP heatmap and integrated analysis of microbial communities and untargeted metabolomics; Figure S4: Supplemental Materials for Figure 6; Figure S5: Supplemental material for Figure 7; Figure S6: ROC for univariate COX regression analysis; Table S1: qPCR primer list.
