# Supplementary material for: Akkermansia muciniphila Metabolite Inosine Inhibits Castration Resistance in Prostate Cancer
Source: Microorganisms. 2024 Aug 12;12(8):1653. doi: 10.3390/microorganisms12081653 (PMC11356635; doi:10.3390/microorganisms12081653)
Supplement: Supplementary file 1 [file microorganisms-12-01653-s001.zip › Supplemental figures.pdf]

Manuscript ID: microorganisms-3132067

Title: *Akkermansia muciniphila* metabolite inosine inhibits castration resistance in prostate cancer

Supplemental figures

Figure S1. Alpha diversity of the gut microbiota in mice and patients.

Figure S2. Serum testosterone level in mice.

Figure S3. Metabolite VIP heatmap and integrated analysis of microbial communities and untargeted metabolomics.

Figure S4. Supplemental material for Figure 6.

Figure S5. Supplemental material for Figure 7.

Figure S6. ROC for univariate COX regression analysis.

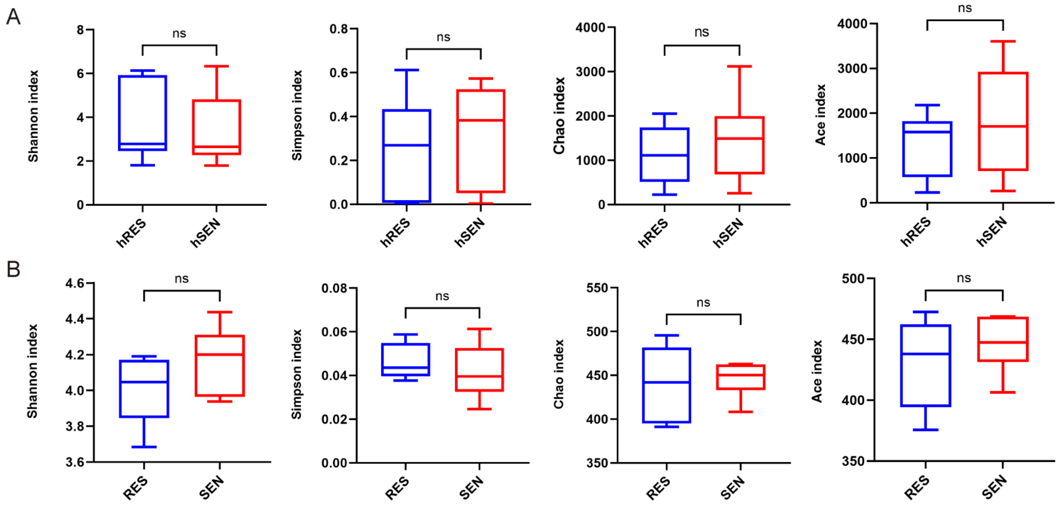

Figure S1. Alpha diversity of the gut microbiota in mice and patients. (A) Alpha diversity of the hRES and hSENgroups patients by Shannon, Simpson, Chao, and Ace index. (B) Alpha diversity of the RES and SEN groups mice by Shannon, Simpson, Chao, and Ace index. Data were expressed as the mean ± SD, ns = no significance.

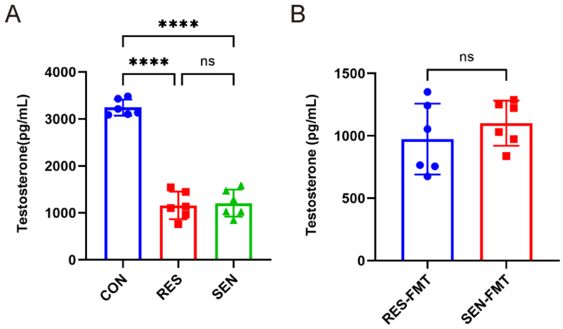

Figure S2. Serum testosterone level in mice. (A) Serum testosterone level in RES and SEN groups mice. n = 6. (B) Serum testosterone level in mice administered RES-FMT or SEN-FMT. n = 6. Data were expressed as the mean ± SD, ns = no significance, ****$p < 0.0001$.

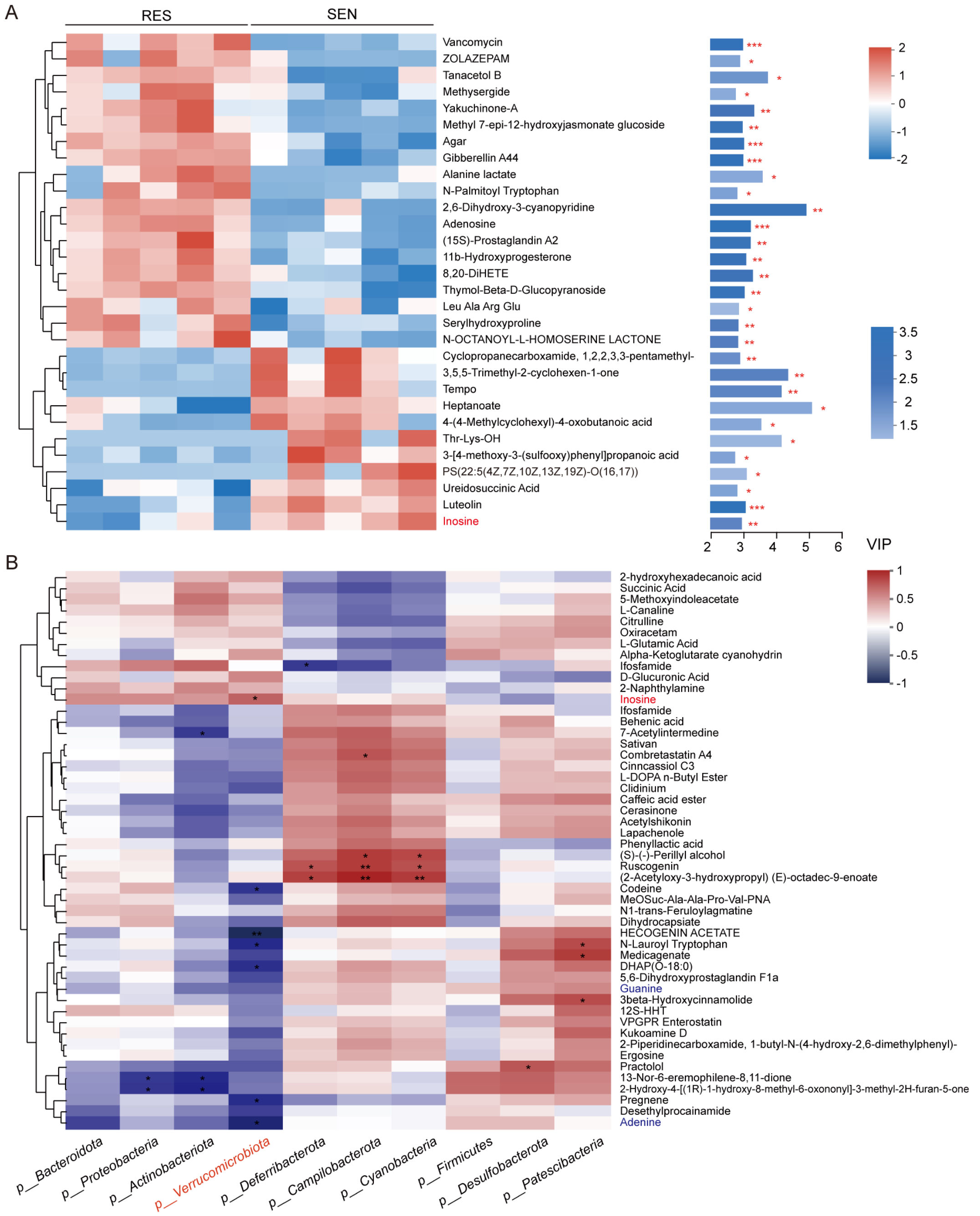

Figure S3. Metabolite VIP heatmap and integrated analysis of microbial communities and untargeted metabolomics. (A) VIP heatmap of differential metabolites between RES and SEN groups. n = 5. (B) Correlation heatmap of integrated analysis of microbial communities and untargeted metabolomics. Data were expressed as the mean ± SD, *$p < 0.05$, **$p < 0.01$, ***$p < 0.001$. VIP, Variable Importance in Projection.

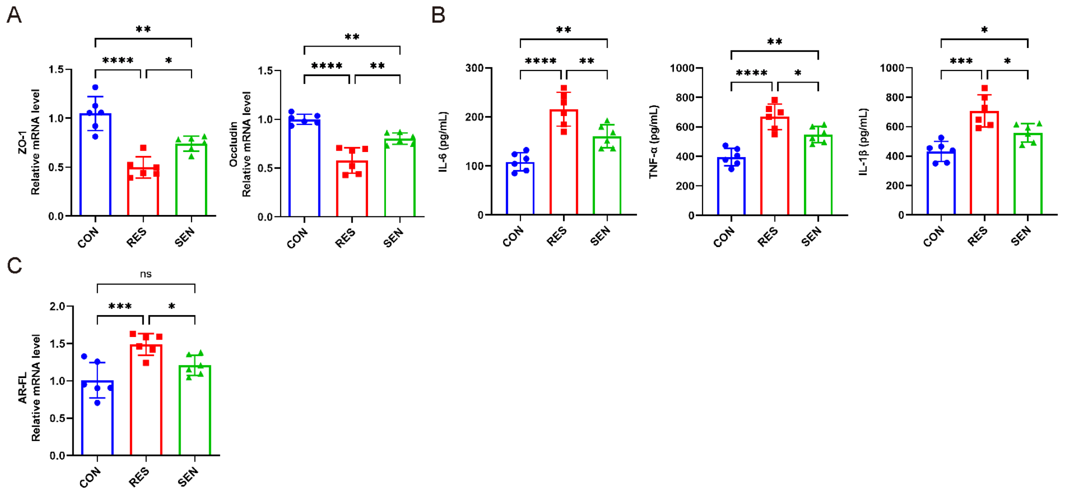

Figure S4. Supplemental material for Figure 6. (A) The transcriptional levels of ZO-1 and Occludin in the colon of RES and SEN groups mice by qPCR. n = 6. (B) Serum IL-6, TNF-α, and IL-1β levels in RES and SEN groups mice. n = 6. (C) The transcriptional level of AR in the tumor of RES and SEN groups mice by qPCR. n = 6. Data were expressed as the mean ± SD, *p < 0.05, **p < 0.01, ***p < 0.001, ****p < 0.0001.

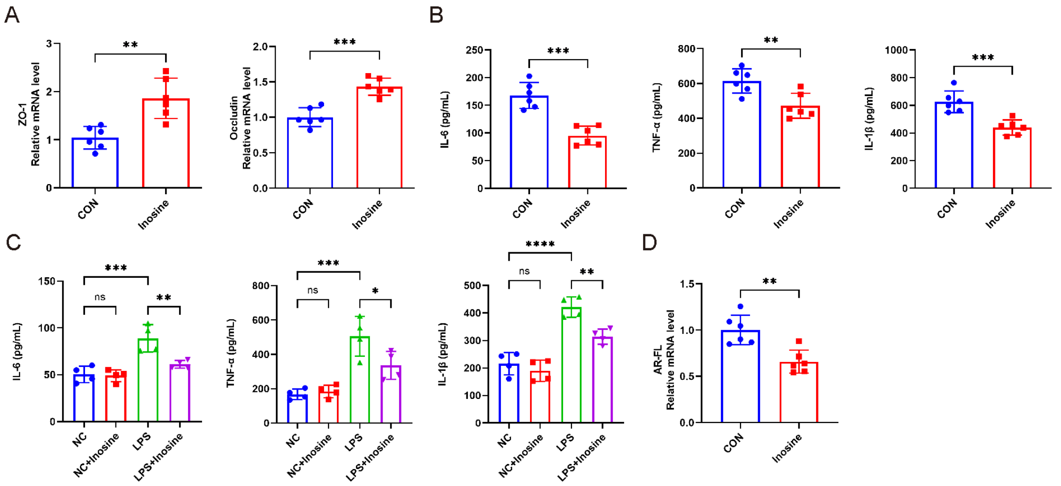

Figure S5. Supplemental material for Figure 7. (A) The transcriptional levels of ZO-1 and Occludin in the colon of mice treated with inosine by qPCR. n = 6. (B) Serum IL-6, TNF-α, and IL-1β levels in mice treated with inosine by qPCR. n = 6. (C) IL-6, TNF-α, and IL-1β levels in Caco-2 culture supernatant after LPS stimulation and inosine intervention. n = 4. (D) The transcriptional level of AR in the colon of mice treated with inosine by qPCR. n = 6. Data were expressed as the mean ± SD, *p < 0.05, **p < 0.01, ***p < 0.001, ****p < 0.0001.

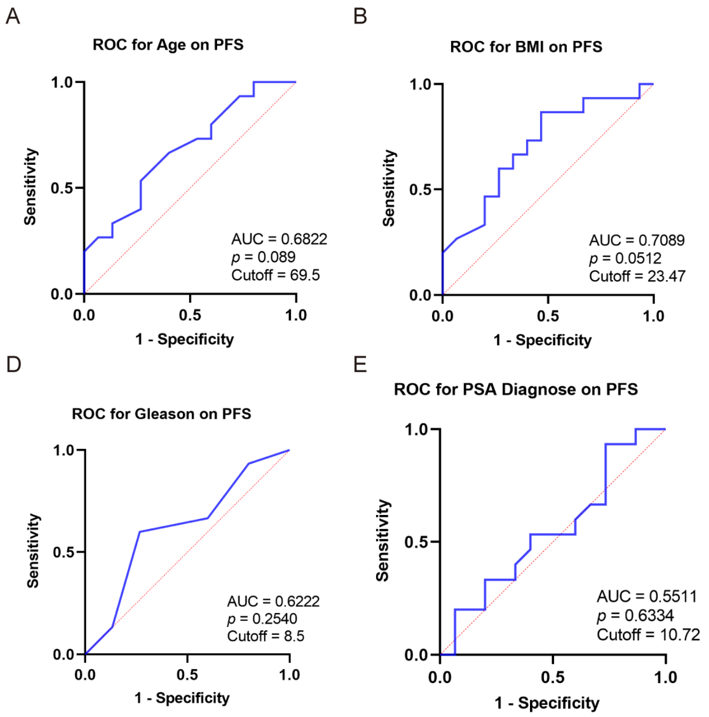

Figure S6. ROC for univariate COX regression analysis. ROC analysis for Age (A), BMI (B), TNM (C), Gleason (D), PSA diagnose (E), and nPSA (F) on PFS. BMI, body mass index; nPSA, nadir of prostate-specific antigen; PFS, progression-free survival.
