# Supplementary material for: Akkermansia muciniphila Metabolite Inosine Inhibits Castration Resistance in Prostate Cancer
Source: Microorganisms. 2024 Aug 12;12(8):1653. doi: 10.3390/microorganisms12081653 (PMC11356635; doi:10.3390/microorganisms12081653)
Supplement: Supplementary file 1 [file microorganisms-12-01653-s001.zip › Supplemetary table.pdf]

Table S1. qPCR primer list.

Gene	Forward primer (5'–3')	Reverse primer (5'–3')
Akkermansia	CTTCGTGCTGGAAATCAACACC	CGATAATTCCGCTATTTTTTCGC
16S	GTGSTGCAYGGYTGTCGTCA	ACGTCRTCCMCACCTTCCTC
ZO-1	AGAGACAAGATGTCCGCCAG	TGCAATTCCAAATCCAAACC
Occludin	ACTCCTCCAATGGCAAAGTG	CCCCACCTGTCGTGTAGTCT
AR-FL	CCTGGCACACTCTCTTCACA	CCGGAGTAGCTATCCATCCA
GAPDH	AGGTCGGTGTGAACGGATTTG	TGTAGACCATGTAGTTGAGGTCA
